# Obituary

**Published:** 1917-11

**Authors:** 


### OBITUARY

Dr. W. A. White, of Phelps, N. Y., died at his home October 15, after a lingering illness of several months. Dr. White was a prominent member of the profession in his own state and made a national reputation as a leader in the propaganda of a few years ago for the education of the public in matters of oral hygiene. He spent much time as a member of the committee of the National Dental Association in organizing the profession for imparting dental hygiene instruction to the public. He lectured for the New York Health Department throughout the state and was a member of the New York State Board of Examiners. He was widely known and admired for his personal qualities of mind and heart. He did his work well and all who knew him will regret his death.

